# Plant traits correlated with generation time directly affect inbreeding depression and mating system and indirectly genetic structure

**DOI:** 10.1186/1471-2148-9-177

**Published:** 2009-07-27

**Authors:** Jérôme Duminil, Olivier J Hardy, Rémy J Petit

**Affiliations:** 1 Université Libre de Bruxelles, Faculté des Sciences, Service Evolution Biologique et Ecologie, CP 160/12, 50 Av. F. Roosevelt, 1050 Bruxelles, Belgium; 2 Faculté Universitaire des Sciences Agronomiques de Gembloux, Laboratoire d'écologie, 2 Passage des Déportés 5030 Gembloux, Belgium; 3 INRA, UMR 1202 Biodiversity, Genes and Communities, F-33610 Cestas, France; 4 Université de Bordeaux, UMR 1202 Biodiversity, Genes and Communities, F-33610 Cestas, France

## Abstract

**Background:**

Understanding the mechanisms that control species genetic structure has always been a major objective in evolutionary studies. The association between genetic structure and species attributes has received special attention. As species attributes are highly taxonomically constrained, phylogenetically controlled methods are necessary to infer causal relationships. In plants, a previous study controlling for phylogenetic signal has demonstrated that Wright's *F*_ST_, a measure of genetic differentiation among populations, is best predicted by the mating system (outcrossing, mixed-mating or selfing) and that plant traits such as perenniality and growth form have only an indirect influence on *F*_ST _via their association with the mating system. The objective of this study is to further outline the determinants of plant genetic structure by distinguishing the effects of mating system on gene flow and on genetic drift. The association of biparental inbreeding and inbreeding depression with population genetic structure, mating system and plant traits are also investigated.

**Results:**

Based on data from 263 plant species for which estimates of *F*_ST_, inbreeding (*F*_IS_) and outcrossing rate (*t*_m_) are available, we confirm that mating system is the main influencing factor of *F*_ST_. Moreover, using an alternative measure of *F*_ST _unaffected by the impact of inbreeding on effective population size, we show that the influence of *t*_m _on *F*_ST _is due to its impact on gene flow (reduced pollen flow under selfing) and on genetic drift (higher drift under selfing due to inbreeding). Plant traits, in particular perenniality, influence *F*_ST _mostly via their effect on the mating system but also via their association with the magnitude of selection against inbred individuals: the mean inbreeding depression increases from short-lived herbaceous to long-lived herbaceous and then to woody species. The influence of perenniality on mating system does not seem to be related to differences in stature, as proposed earlier, but rather to differences in generation time.

**Conclusion:**

Plant traits correlated with generation time affect both inbreeding depression and mating system. These in turn modify genetic drift and gene flow and ultimately genetic structure.

## Background

One achievement of empirical population genetic studies is the survey of the genetic diversity of thousands of plant and animal species using molecular markers, first on the basis of proteins and then of DNA. This "immense outpouring of data on genetic variation" has been considered to be both a milestone, because any type of organism could be investigated using standardised molecular methods, and a "millstone around our neck", because it resulted in the "depauperization of the diversity of empirical work in evolutionary genetics" [1]. Notwithstanding possible side-effects on the development of the field, such a large dataset of species-level microevolutionary approaches should allow addressing important issues in comparative biology. However, few studies have taken advantage of this rich but heterogeneous data for drawing inferences on patterns of genetic variation and on their relation with species attributes. Among the few attempts to relate genetic diversity statistics to species attributes, some influential studies have dealt with plants (e.g. [2-5]). Unfortunately, these early studies neither accounted for phylogenetic inertia in the data nor for potentially confounding covariates, making it difficult to identify causal mechanisms. We recently showed that when accounting for phylogenetic signal and covariation between traits, the only species trait that consistently predicted genetic structure was the mating system: selfing species tend to build up more genetic structure than outcrossing species [6]. Mating system was also shown to be the best predictor of plant genetic diversity [7]. However, mating system was not quantified precisely in these studies [2-4,7]. Furthermore, plant traits could affect genetic structure indirectly, via their effect on mating system, so predicting mating system itself on the basis of plant traits is of interest. Here we revisit the interrelations between population genetic structure, mating system and plant traits using a quantitative estimate of the mating system (i.e. outcrossing rate), while controlling phylogenetic signal and using traits as covariates.

Species genetic structure reflects the balance between divergence processes and cohesion processes among populations. In particular, gene flow limits genetic structure, whereas genetic drift increases genetic structure. Hence, traits affecting mating system and dispersal should affect genetic structure by modifying the drift/migration equilibrium. Since traits affecting genetic structure often evolve conservatively during evolution, measures of genetic structure typically present a strong phylogenetic signal (see e.g. [6]). This does not imply that *F*_ST _[8], which measures the mean proportion of the total genetic variance contained in a population, is an intrinsically 'heritable' feature of species. In fact, it is expected to approach equilibrium on a relatively short time scale [9], compared to speciation events. Its strong phylogenetic inertia is due to the fact that related species share similar traits influencing the distribution of genetic diversity. Once phylogeny is controlled for when testing relationships between plant traits and genetic structure, *F*_ST _(as measured at biparentally-inherited nuclear markers) has been shown to depend mostly on the inbreeding coefficient (*F*_IS_) and the mating system, whereas life form or perenniality appear to affect genetic structure only indirectly, through their effects on the mating system and inbreeding [6].

Mating system and inbreeding have long been known to influence species population genetic structure [2,4,6,10,11]. But only qualitative classifications of plant mating systems (selfed, mixed and outcrossed) have been used to study this relationship [2,4,6]. The classification of mating system on the basis of floral morphology can lead to false attributions and does not provide any quantitative measure of actual outcrossing rates. Direct analyses of offspring genotypic data at several gene loci allow a much more precise quantification of multilocus outcrossing rates *t*_m_[12]. Such quantitative measures of mating system should help study the association between plant traits, mating system, inbreeding and *F*_ST_.

Mating system can affect the distribution of genetic diversity in different ways, in particular by its effect on pollen-mediated gene flow and by its effect on genetic drift within population. This is because selfing can (i) lower gene flow among populations by limiting pollen-mediated gene flow (as pollen used for self-pollination does not contribute to gene flow) and (ii) increase inbreeding, thereby reducing effective population size *N*_e _and increasing genetic drift [13]. Which of these two effects predominates in plants is typically not known. The definition of an alternative measure of *F*_ST _not affected by the reduction of *N*_e _due to inbreeding would help to evaluate their respective influence.

Mating system is related with inbreeding in a complex, interdependent way: while outcrossing rate should have a strong impact on inbreeding, this factor, in turn, directly influences the evolution of the mating system. Indeed, the main factor controlling mating system evolution appears to be inbreeding depression, the detrimental effects of close inbreeding on fitness [14]. In plants, inbreeding depression affects inbred (especially selfed) progeny, particularly during the earliest stages of the plant's life cycle [15]. Hence, the inbreeding coefficient (*F*_IS_) of adult plants integrates information not only on selfing rate [16] but also on inbreeding depression. The selective removal of inbreds limits genetic drift and hence indirectly genetic structuring. When pollen and seed dispersal are limited, resulting in intra-population structure, *F*_IS _can also be affected by biparental inbreeding. Hence, comparing the expected inbreeding coefficient due to selfing, *F*_e_, with the *F*_IS _observed at the adult stage allows testing the impact of both inbreeding depression and biparental inbreeding on population genetic structure.

Inbreeding depression could depend on plant traits and is typically stronger in perennials than in annuals [17-19]. Among the models proposed to explain the relationships between plant traits and inbreeding depression, the Τ-model of plant mating system evolution states that the per-generation mutation rate of a plant is a function of the number of mitoses that occur from zygote to gamete. High-Τ plants (typically, high-stature plants) should accumulate more mutations during their lifetime and their offspring should therefore show more inbreeding depression than low-Τ plants (low-stature plants) [17], thereby selecting for outcrossing. Alternatively, mating system could depend not so much on the per-generation mutation rate but on generation time per se [20]. This is expected if inbreeding depression had particularly detrimental effects in long-lived plants (for instance because it has multiplicative effects across years, [21]). Finally, generation time rather than stature could drive mating system evolution if the survival of short-lived plants but not of long-lived ones would depend on reproductive assurance conferred by selfing.

In this study, we have gathered data on the partitioning of diversity within and among populations (*F*_ST_), on individual inbreeding (*F*_IS_), on the mating system (based on a quantitative estimate, the outcrossing rate *t*_m_), and on some key plant traits (stature, growth form, perenniality, mode of pollen dispersal) for 263 plant species. A characterization of the correlation structure among these variables using phylogenetically-controlled regression methods should provide insights into the determinants of plant genetic structure. In addition, using an alternative to *F*_ST _that controls for the impact of inbreeding on genetic differentiation, we will attempt to distinguish the effects of the mating system on gene flow and on genetic drift. Finally, we will test the occurrence of biparental inbreeding and of selection against inbreds to assess their relevance in explaining the relationships between population genetic structure, mating system and plant traits.

## Methods

### The database

A systematic search in the peer-reviewed literature [17,22] yielded 263 plant species for which the following information were available: (i) *genetic structure *at nuclear markers, as measured by the *F*_ST _index, (ii) inbreeding (or *heterozygote deficit*), as measured by the *F*_IS _index, (iii) *mating system*, as measured by the multilocus outcrossing rate *t*_m _(additional file 1). Estimates of outcrossing rate that exceeded 1 (which is technically possible using unbiased estimators) were set to 1 because all reported values did not use the same method of estimation. *F*_ST _estimates were based on allozymes for 216 species and on nuclear microsatellites for 33 species. Although the higher mutation rate at microsatellite loci might sometimes violate the assumption that mutation is negligible relative to migration [23], no difference in *F*_ST _due to the type of marker was detected (results not shown).

### An alternative measure of *F*_ST _not affected by the reduction of *N*_e _due to inbreeding

In a neutral island model, *F*_ST_/(1-*F*_ST_) = 1/(4*N*_e_.*m*) at equilibrium, where *m *is the migration rate among populations. Therefore, selfing is expected to increase *F*_ST _for at least two reasons: (i) selfing can reduce *m *by limiting pollen-mediated gene flow among populations (in strictly selfing species, only seed dispersal contributes to the overall gene dispersal), and (ii) as a consequence of inbreeding, selfing should reduce *N*_e _by a factor of 1/(1+*F*_IS_) [13]. Hence, when the within-population heterozygote deficit *F*_IS _is positive, *N*_e _= *N*_e_'/(1+*F*_IS_), where *N*_e_' is the expected effective population size in the absence of inbreeding (*N*_e_' is the reciprocal of the probability that two gametes contributing to random separate adults come from the same parent). To distinguish the two effects of selfing on genetic structure, we define an alternative measure, *F*_ST_' = *F*_ST_/[(1+*F*_IS_)(1-*F*_ST_)], closely related to the parameter *ρ *[24]. Like *ρ*, *F*_ST_' is not affected by the reduction of *N*_e _due to inbreeding because *F*_ST_'/(1-*F*_ST_') = 1/(4*N*_e_'.*m*) in an island model at equilibrium. Hence, under a neutral model, selfing will increase *F*_ST_' only if it reduces *m *by limiting pollen-mediated gene flow.

### Plant traits

For each species (additional file 1), information on the following traits was gathered (either from the original publications, by contacting directly the authors of the original articles, in floras or by searching in specialised web sites):

• *Species stature*, i.e. plant size, expressed in meters. We expect a positive relationship between the stature of the plant and the dispersal ability (through pollen and seeds).

• *Species growth form*, classified as woody or herbaceous. Succulent species like *Hedysarum carnosum *and *Melocactus curvispinus*, which are rare in our database, were grouped with woody species.

• *Species perenniality*, corresponding to the distinction between short-lived species (annual and biennial species) and long-lived species (perennials). Note that growth form and perenniality, although strongly correlated (all woody plants are perennials), group different species, since herbaceous plants can be either short-lived or long-lived. Those species described in the literature as either short-lived or long-lived species (e.g. depending on the environment) were classified as long-lived species.

• *Species mode of pollen dispersal*, coded as biotic (insects, bats, birds) or abiotic (wind or water). Both biotic and abiotic pollen dispersal were described for *Cocos nucifera *and for *Castanea sativa*; they were grouped together with species having abiotic mode of pollination.

The analysis of the relationship between genetic structure (or mating system) and plant traits depends on the identification of the most relevant traits. In this respect, it would have been preferable to include generation time rather than some of its surrogates such as perenniality in the analyses. Similarly, the assignment of mode of pollination into discrete categories (abiotic and biotic) can be criticised as it is not always based on field observations and ignores the potential for mixed dispersal factors [25]. However, such information is generally not available in the literature.

### Phylogenetic tree

A phylogenetic supertree (additional file 2) was built using as backbone (up to the family level) the updated version of the plant phylogenetic tree published by the angiosperm phylogeny group [26]. The details of the topology at lower taxonomic levels were then grafted according to phylogenetic information obtained from various sources, generally from specific phylogenetic studies at the family level or below [27-45]. Nevertheless some phylogenetic relationships were left as soft polytomies when it was not possible to resolve the topology due to lack of published information. Given the different sources of phylogenetic information it was not possible to determine branch lengths which were therefore set to 1, an assumption that generally performs well for phylogeny-controlled comparative studies [46].

### Statistical analyses

#### Transformation of the variables

Qualitative variables were coded as dummy: for growth form, herbaceous = 0 and woody = 1; for perenniality, short-lived = 0 and long-lived = 1; for mode of pollen dispersal, abiotic = 0 and biotic = 1. To improve normality of the continuous variables (*F*_ST_, *F*_ST_', *F*_IS_, *t*_m _and stature) we used the option "Box-Cox-Bartlett transformations" included in the R Package for Multivariate and Spatial Analysis Version 4.0 [47].

#### Transformation of the data into phylogenetically-independent contrasts

To control for the phylogenetic relationships among species, data were transformed into phylogenetically independent contrasts (PICs, [48]) using the PDAP module [49] included in Mesquite software [50]. Phylogeny controlled methods are statistically conservative and tend to perform better in cross-species comparisons than non-phylogenetically controlled methods, even when their assumptions are violated (like the non-Brownian mode of evolution of the characters or inconsistencies of the chosen phylogenetic hypothesis) [51].

#### Regression analyses

Regression analyses were conducted under the following assumptions, based on population genetics theory. (i) The outcrossing rate (*t*_m_) can be influenced by plant traits. (ii) Individual inbreeding within population (*F*_IS_) can be influenced by plant traits and by *t*_m_. (iii) Genetic differentiation (*F*_ST _and *F*_ST_') can be influenced by plant traits, *t*_m_, and *F*_IS_. Regressions analyses were performed on the raw data (TIPs analyses: comparison of the values taken at the tips of the tree) and on the independent contrasts using SYSTAT, version 10.2.05 [52]. For PICs analyses, regressions were forced through the origin [48]. Multiple regression analyses were performed with the forward stepwise regression option to integrate in the model the independent variables as a function of their importance (i.e. their degree of relatedness with the dependent variable).

Species were considered mixed mating species when 0.1 <*t*_m _< 0.9 (number of species *N *= 129) or outcrossed species when *t*_m _> 0.9 (*N *= 129). Only five selfed species (*t*_m _< 0.1) were present in the database, so no selfed category was used.

### Assessing biparental inbreeding and inbreeding depression

For the subset of non-outcrossed species for which both *t*_m _and *t*_s _(*t*_s _is the mean of single locus outcrossing rate) were available (80 species), we apply a sign test on the difference *t*_m _-*t*_s _to check for the presence of biparental inbreeding. A positive difference (*t*_m _>*t*_s_) will demonstrate the presence of biparental inbreeding as *t*_s _should be more affected by biparental inbreeding than the multi-locus estimator *t*_m _(due to an over-estimation of the selfing rate when polymorphism is low, [12]).

The expected inbreeding *F*_e _would correspond to the fixation index (*F*_IS_) if selfing were the sole factor causing deviation from Hardy-Weinberg equilibrium. For a mixed mating model in the absence of inbreeding depression, *F*_e _is estimated as: *F*_e _= (1-*t*_m_)/(1+*t*_m_) [16]. Differences in the levels of inbreeding coefficient between seed and adult cohorts would suggest that other factors than selfing influence genotypic frequencies [53]. High *F*_IS _estimates relative to the expectation could be caused by biparental inbreeding, whereas low estimates could reflect the impact of inbreeding depression. To test for statistical trends in the distribution of *F*_e _- *F*_IS _for all species and then separately for each growth form by perenniality category (short-lived herbaceous, long-lived herbaceous and woody species), we applied a sign test over all species except outcrossed ones. Predominantly outcrossed species were excluded from these analyses because the seeds produced are not inbred, so that inbreeding depression, even if it exists, cannot manifest itself.

Under inbreeding depression (δ ≡ 1-w_i_/w_o _where w_i _and w_o _are the fitness of inbred and outbred individuals, respectively), the expected inbreeding is [53]:

(1)

For each growth form by perenniality category, an average value of δ was estimated by least square fitting of this equation on the observed *F*_IS _and *t*_m _values. This approach neglects inter-species variation but captures general trends of the intensity of inbreeding depression according to growth form and perenniality.

## Results

### Taxonomic coverage

The 263 species are distributed into 155 genera and 73 families; there are 63 gymnosperms genera (all conifers) and 92 angiosperms genera (seven magnoliids, 22 monocots, 63 eudicots). Four botanical families are particularly represented (families that contain more than 5% of the species included in the database): Asteraceae, Fabaceae, Myrtaceae (mainly the genus *Eucalyptus*) and Pinaceae; together they include 47% of the species of the data set.

### General patterns

**Figure 1:**
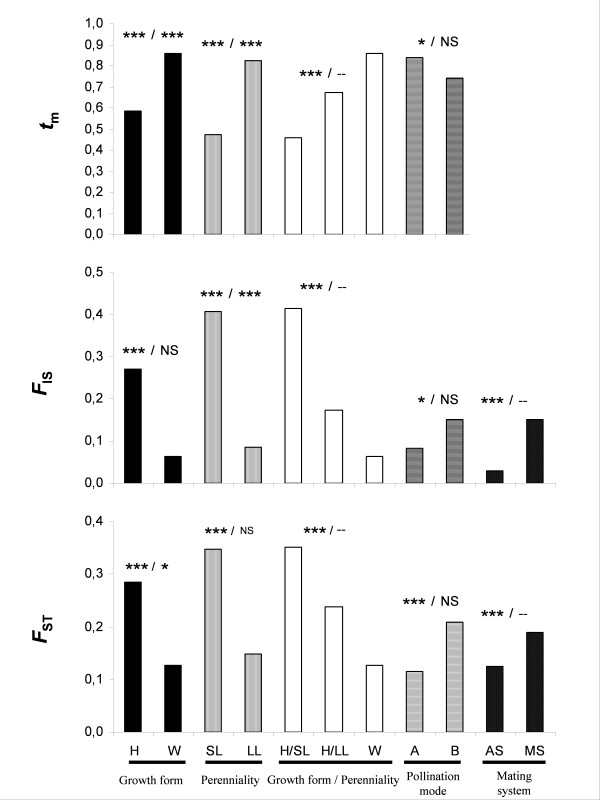
**Mean values of *t*_m_, *F*_IS _and *F*_ST _as a function of growth form (H: herbaceous, W: woody), perenniality (SL: short-lived, LL: long-lived), the combination of growth form and perenniality (H/SL: herbaceous short-lived, H/LL: herbaceous long-lived, W/LL: woody long-lived), the mode of pollen dispersal (A: abiotic, B: biotic) and outcrossing rate (MS: mixed mating species, AS: allogamous species). **For each category the *P*-value associated with the statistical test is indicated by *** *P *< 0.001, ** 0.001<*P *< 0.01, * 0.01<*P *< 0.05 and ^NS ^for *P *> 0.05. At the left part of the slash the *P*-value corresponds to the TIPs test and at the right to the PICs test. "--" indicates an absence of statistical test. Standard error values for each category is available in the additional file 3.

**Figure 2:**
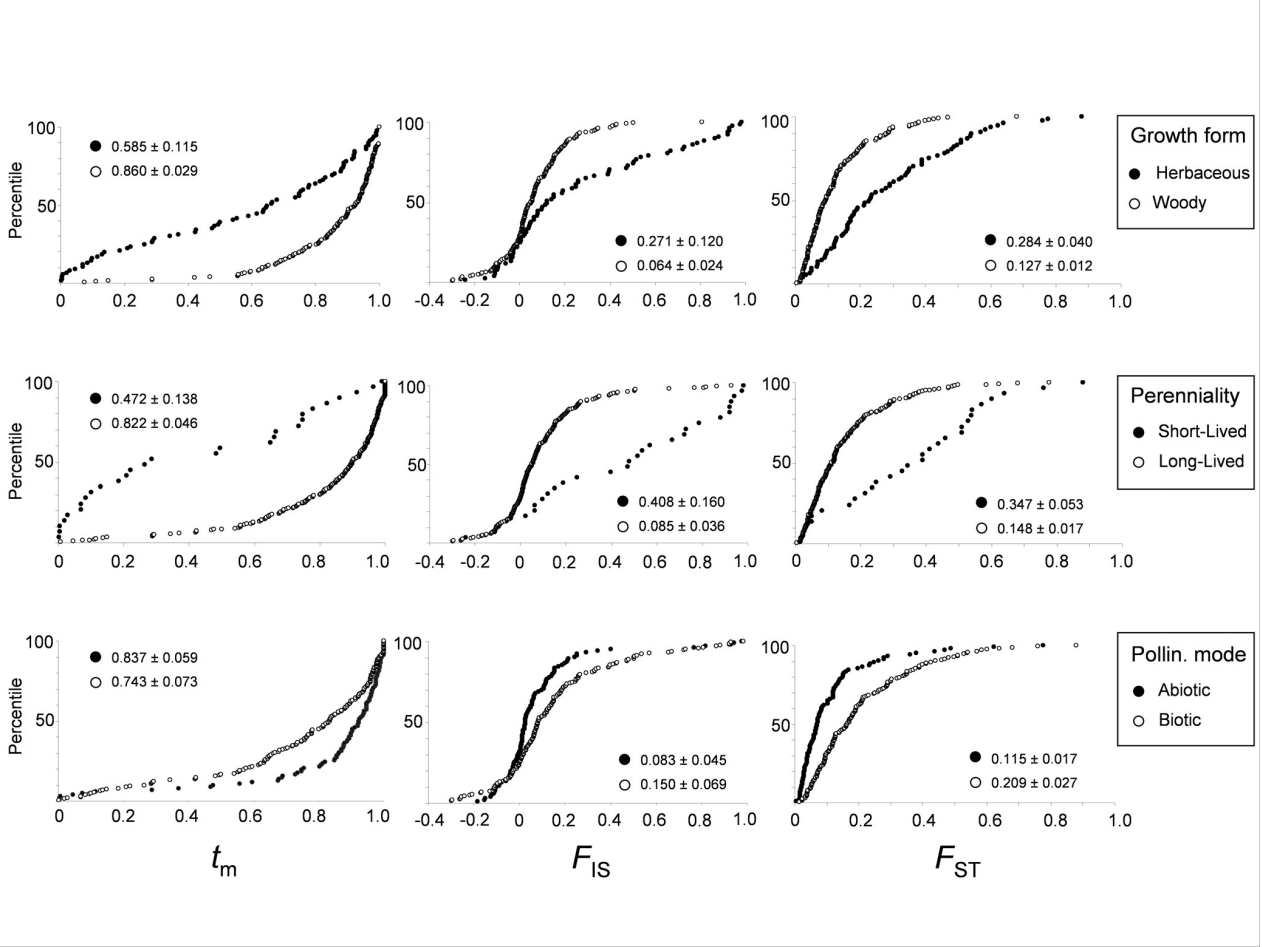
**Distribution of *t*_m_, *F*_IS _and *F*_ST _according to growth form, perenniality and pollination mode categories (percentile versus ranked *t*_m_-estimate, *F*_IS_-estimate and *F*_ST_-estimate data)**.

The mean values of *t*_m_, *F*_IS _and *F*_ST _as a function of growth form, perenniality, a combination of perenniality/growth form and pollination mode are provided in Figure 1 and 2 (and see additional file 3). The distribution of *t*_m_, *F*_IS _and *F*_ST _clearly depends on plant traits. This is particularly remarkable for *t*_m_: long-lived woody species are generally outcrossed, in contrast to short-lived herbaceous species, for which the mating system ranges from fully selfing to fully outcrossing. Values of *F*_ST_, *F*_IS _and *t*_m _covary: when *F*_ST _decreases (for example *F*_ST _is lower for woody species than for herbaceous species), then *F*_IS _also decreases (woody species present a lower inbreeding coefficient than herbaceous species) while *t*_m _increases (woody species are more outcrossed than herbaceous species). Nevertheless most of the statistically significant differences among categories in TIPs analyses disappear when the phylogenetic signal is controlled for (PICs analyses). The only relationships that remain significant are those between growth form and *F*_ST_, between perenniality and *F*_IS_, and between growth form/perenniality and *t*_m_.

### Relationships among traits

**Figure 3:**
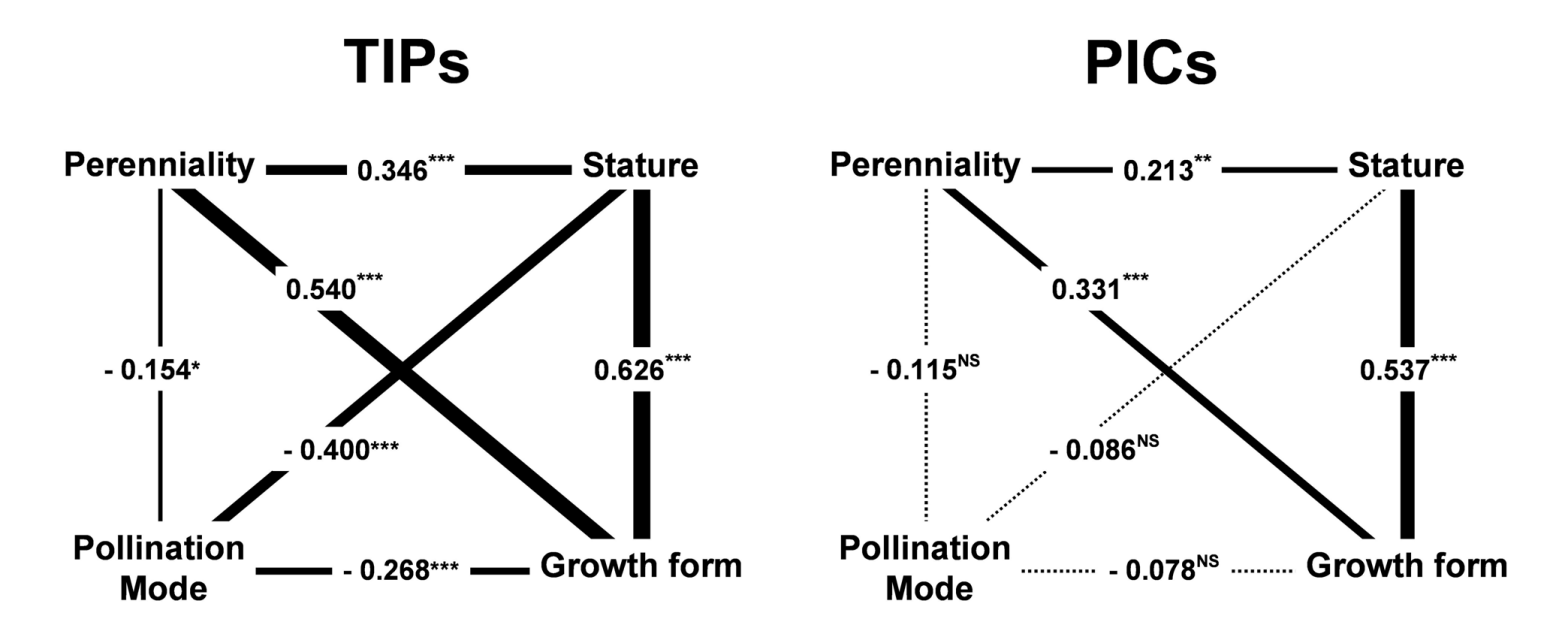
**Conventional regressions analyses (TIPs) on the left and phylogenetically-controlled regression analyses (PICs) on the right, among plant traits: growth form, perenniality, pollination mode and stature**. *** *P *< 0.001, ** 0.001<*P *< 0.01, * 0.01<*P *< 0.05 and ^NS ^for *P *> 0.05.

Stature, growth form and perenniality were found to be positively associated using both TIPs and PICs analyses (Figure 3). In contrast, TIPs but not PICs analyses identify significant relationships between pollination mode and the other traits, which are thus likely due to the phylogenetic conservatism of the studied traits.

### Relationships between traits and *F*_ST _(figure 1, table 1 and additional file 4)

**Table 1:** Phylogenetically-controlled regression analyses between *t*_m_, *F*_IS_, *F*_ST_, *F*_ST_' and other variables

	***t***_m_		***F***_IS_		***F***_ST_		***F***_ST_'	
				
	R ^‡^	ΔR^2 ^^†^	R ^‡^	ΔR^2 ^^†^	R ^‡^	ΔR^2 ^^†^	R ^‡^	ΔR^2 ^^†^
				
*t*_m_	**NC**		-0.511***	0.261	-0.343***	0.034	-0.272***	0.073
*F*_IS_	**NC**		**NC**		0.361***	0.130	0.213***	--
Stature	0.203***	--	-0.059^NS^		-0.135*	--	-0.134*	--
Growth form^a^	0.339***	0.054	-0.115^NS^		-0.146*	--	-0.138*	--
Perenniality^b^	0.362***	0.131	-0.293***	0.013	-0.115 ^NS^		-0.081^NS^	
Pollination mode^c^	0.019^NS^		-0.004^NS^		0.041 ^NS^		0.042^NS^	

*** *P *< 0.001, ** 0.001<*P *< 0.01, * 0.01<*P *< 0.05 and ^NS ^for *P *> 0.05. NC indicates variables not considered as explanatory variable.

^a ^Herbaceous species were coded 0 and woody species 1; ^b ^Short-lived species (annuals and biannuals) were coded 0, and long-lived species (perennial species) were coded 1; ^c ^The mode of pollination was coded 0 for abiotic (wind- or water-dispersed) and 1 for biotic (animal-dispersed)

^‡ ^correlation coefficient of simple regression analyses

^† ^multiple regression analyses (stepwise option using all significant variables from the simple regression analyses): ΔR^2 ^represents the gain of R^2 ^(proportion of variation explained by the variables) when the variable is incorporated into the model, "--" indicates that the variable does not enter in the model, after the integration of the most explicative variables.

All four traits studied are strongly related with F_ST _in TIPs analyses (regression coefficient of F_ST _as a function of stature is -0.45, P < 0.001). Herbaceous, short-lived, low-stature, biotically-pollinated, mixed-mating species present higher levels of genetic structure than woody, long-lived, high-stature, abiotically-pollinated, allogamous species. The decomposition of herbaceous species into short-lived and long-lived species demonstrates the impact of the perenniality within herbaceous species, as short-lived herbaceous species present higher F_ST _than long-lived herbaceous species (figure 1). When the phylogenetic signal is controlled for, F_ST _depends on F_IS_, t_m_, growth form and species stature (table 1). F_ST _increases when F_IS _increases and decreases when t_m _increases. F_ST _is negatively related to plant stature, and herbaceous species have lower F_ST _than woody species. The pollination mode has no influence on F_ST _(table 1). In long-lived species, F_ST _is also negatively associated with t_m _and positively with F_IS _and with species stature, whereas long-lived woody species have lower F_ST _than long-lived herbaceous species (additional file 4). In a multiple regression framework (PICs, table 1), variation in t_m _and F_IS _accounts for variation in F_ST _but the growth form and the stature do not enter into the model once t_m _and F_IS _are included. Additional file 4 indicates that for herbaceous and woody species the main influencing factor is F_IS _(the effect of t_m _disappears in the multiple regression).

### Relationships between traits and F_ST_' (table 1 and additional file 4)

As for *F*_ST_, *F*_ST_' depends on *F*_IS_, *t*_m_, stature and growth form (table 1). The strength of the relationship between *F*_ST_' and *t*_m _and between *F*_ST_' and *F*_IS _is lower than the respective relationships with *F*_ST_. Contrarily to *F*_ST_, in a multiple regression with stepwise selection, *t*_m _is the main explanatory variable of *F*_ST_' and *F*_IS _does not enter the model once *t*_m _is accounted for. The comparison between *F*_ST _and *F*_ST_' is particularly interesting when focusing on species from different categories (long-lived, herbaceous, woody, additional file 4). In long-lived species, *F*_ST_' is no longer associated with *F*_IS_. By contrast *F*_ST_' is still related with *F*_IS _in herbaceous species, although *t*_m _remains the main explanatory variable.

### Relationships between traits and FIS (figure 1, tables 1 and 2)

**Table 2:** Phylogenetically-controlled regression analyses between *F*_IS _and other variables

	Long lived species		Herbaceous		Woody	
			
Variable	R ^‡^	ΔR^2 ^^†^	R	ΔR^2 ^^†^	R	ΔR^2 ^^†^
*t*_m_	-0.354***		-0.780***	0.608	-0.276***	
Stature	0.004^NS^		-0.192^NS^		0.022^NS^	
Growth form ^d^	-0.028^NS^		**NC**		**NC**	
Perenniality ^e^	**NC**		-0.410***	--	**NC**	
Pollination mode ^f^	-0.058^NS^		0.094^NS^		-0.045^NS^	

Same legend as table 1.

All traits are correlated with the inbreeding coefficient *F*_IS_in TIPs analyses (figure 1; regression coefficient *F*_IS _as a function of stature is -0.27, *P *< 0.001). Herbaceous, short-lived, low-stature, biotically-pollinated, mixed-mating species present higher inbreeding coefficient than, respectively, woody, long-lived, high-stature, abiotically-pollinated, allogamous species. Moreover, short-lived herbaceous species present higher *F*_IS _than long-lived herbaceous species (figure 1). Based on PICs analyses, the inbreeding coefficient depends mainly on the outcrossing rate, with outcrossed species displaying lower inbreeding (table 1). But perenniality also remains a statistically significant predictive factor of the level of *F*_IS_, with perennial species characterised by lower inbreeding. By contrast, there is no residual relationship between the size of the plants and *F*_IS_. The outcrossing rate *t*_m _is negatively associated with *F*_IS _for all categories of plants (long-lived, herbaceous and woody species, table 2). In addition, for herbaceous species, there is an effect of perenniality, but it does not persist when *t*_m _is accounted for (table 2).

### Relationships between traits and outcrossing rate tm (figure 1, table 1 and 3 and additional file 5)

**Table 3:** Phylogenetically-controlled regression analyses between *t*_m _and other variables

	Long lived species^a^		Herbaceous^b^		Woody^c^	
			
Variable	R ^‡^	ΔR^2 ^^†^	R	ΔR^2 ^^†^	R	ΔR^2 ^^†^
Stature	0.162*	--	0.152^NS^		0.029^NS^	
Growth form ^d^	0.239***	0.057	**NC**		**NC**	
Perenniality ^e^	**NC**		0.423***		**NC**	
Pollination mode ^f^	0.082^NS^		0.027^NS^		0.020^NS^	

^a ^N = 230 ^b ^N = 76 ^c ^N = 185

0.01<*P *< 0.05 and ^NS ^for *P *> 0.05.

^d ^Herbaceous species were coded 0 and woody species 1; ^e ^Short-lived species (annuals and biannuals) were coded 0, and long-lived species (perennials species) were coded 1; ^f ^The mode of pollination was coded 0 for abiotic (wind- or water-dispersed) and 1 for biotic (animal-dispersed)

^‡ ^correlation coefficient of simple regression analyses

^† ^multiple regression analyses (stepwise option using all significant variables from the simple regression analyses): ΔR^2 ^represents the gain of R^2 ^(proportion of variation explained by the variables) when the variable is incorporated into the model, "--" indicates that the variable does not enter in the model, after the integration of the most explicative variables.

The outcrossing rate is positively associated with all four traits studied, although less strongly with pollination mode (figure 1; regression coefficient of *t*_m _as a function of stature is 0.44, *P *< 0.001, in TIPs analyses). Woody, long-lived, high-stature, abiotically-pollinated species have higher outcrossing rates than herbaceous, short-lived, low-stature, biotically-pollinated species. The associations of *t*_m _with growth form and perenniality persist in PICs analyses, but not that with pollination mode, indicating that previously identified relationships between mating system and pollination mode might be due to pseudoreplication [22] (table 3). Additionally, taller species have higher outcrossing rates (table 1), but not if we restrict the analyses to woody plants (table 3). Based on multiple regression analyses, the strongest relationship was found between mating system and perenniality: annual or biannual species present lower outcrossing rates than perennial species (table 1). Once this variable is taken into account, growth form is still an explanatory variable of *t*_m _despite the fact that most herbaceous species are short-lived species and that all woody species are long-lived species. The additional information stems probably from the difference between herbaceous and woody perennials. To summarize, as already mentioned by [20], average *t*_m _increases in the following sequence: short-lived herbaceous species, long-lived herbaceous species, woody species (figure 1).

### Presence of biparental inbreeding

A positive signal of biparental inbreeding over all species is revealed by the sign test applied on the difference *t*_m_- *t*_s _(124 cases with *t*_m_*> t*_s_, versus only 33 with *t*_m_*< t*_s_, *P *< 0.001). Few informative data are available for short-lived herbaceous species, making it difficult to test for a trend (only five cases, all with *t*_m_*> t*_s_, *P *= 0.063). For perennial species the trend is significant (for herbaceous species: 16 cases versus two with *t*_m_*> t*_s_, *P *= 0.001 and for woody species: 45 versus 10 with *t*_m_*> t*_s_, *P *< 0.001). Differences in levels of biparental inbreeding among categories are not significant (data not shown).

### Difference between observed and predicted inbreeding coefficient

**Figure 4:**
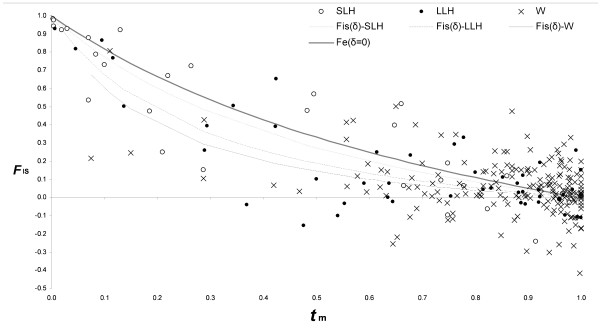
**Distribution of *F*_IS _according to *t*_m_**. The open circles correspond to short-lived herbaceous species, the closed circles to long-lived herbaceous species and the crosses to woody species. The thick continuous line represents *F*_e_, which is the expected *F*_IS _based on the observed *t*_m_, assuming a mixed-mating system at equilibrium without inbreeding depression. The other lines correspond to the best-fitting curves according to equation 1 after adjusting the level of inbreeding depression, δ, for short-lived herbaceous species (short-hatched line), perennial herbaceous species (long-hatched line) and woody species (continuous line).

Figure 4 illustrates the observed inbreeding compared to that expected at equilibrium if selfing was the sole cause of inbreeding (*F*_e _= [1-*t*_m_]/[1+*t*_m_]). There is a global trend towards *F*_IS_*< F*_e _over all species, which indicates a selection against inbred individuals (94 cases, compared to 48 with *F*_IS_*> F*_e_, *P *< 0.001). In short-lived herbaceous species, *F*_IS _is very close to *F*_e_, whereas for perennial species a majority of species have *F*_IS _<*F*_e_, especially when 0.1 <*t*_m _< 0.9 (i.e. in mixed-mating species). There was no significant difference between *F*_IS _and *F*_e _in short-lived species (16 cases with *F*_IS_*< F*_e_, compared to 10 with *F*_IS_*> F*_e_, *P *= 0.33), in contrast to herbaceous perennial species that present a marginally significant trend (23 cases with *F*_IS _*< F*_e_, compared to 11 with *F*_IS_*> F*_e_, *P *= 0.057) and woody species that present a significant trend (55 cases with *F*_IS_*< F*_e_, compared to 27 with *F*_IS_*> F*_e_, *P *= 0.003). However, the mean of *F*_IS_- *F*_e _does not differ significantly between short-lived (*M *= -0.04, *N *= 26) and long-lived species (*M *= -0.08, *N *= 116) (*P *= 0.30).

### Estimation of inbreeding depression

The measures of inbreeding depression δ obtained by least square fitting of the data using the equation [53] are 0.24 for short-lived herbaceous, 0.53 for long-lived herbaceous and 0.66 for woody species. These values are probably somewhat underestimated, given that biparental inbreeding exists in all plant categories; its effect should be to inflate *F*_IS _compared to the equilibrium expectation (*F*_e_) based on selfing rate.

## Discussion

Using for the first time quantitative estimates of outcrossing rate (*t*_m_), we confirm previous studies showing that mating system largely controls plant population genetic structure [2,4,6,54]. Our results, which rely on the use of independent contrasts, also show that once mating system and inbreeding are accounted for, plant traits are no longer associated with *F*_ST _[6]. We further show that mating system affects *F*_ST _via its effect on pollen-mediated gene flow and on effective population size (i.e. drift). These two mechanisms have long been known to affect *F*_ST _[55] but it has generally not been possible to tell them apart. We were able to partially disentangle their effects by comparing traditional *F*_ST _with an alternative parameter (*F*_ST_') that controls for the influence of inbreeding on effective population size but is not affected by the impact of the mating system on inbreeding. Under a neutral model, the negative correlation of *F*_ST_' with *t*_m _indicates that selfing reduces pollen-mediated gene flow among populations. Hence, selfing results not only in increased genetic drift due to inbreeding but also in decreased pollen-mediated gene flow, as discussed by Ingvarsson [56]. However, *F*_ST_' is not significantly correlated with *t*_m _when the analysis is restricted to long-lived species or to woody species, probably because most species are then predominantly outcrossed and a reduction of pollen flow due to selfing becomes hardly perceptible.

Selfing can also reduce *N*_e _by other processes than inbreeding, for instance through selective sweeps and hitchhiking, as a consequence of background selection, or because it magnifies the consequences of extinction and recolonisation [56,57]. This could explain the remaining association between *F*_ST_' and *F*_IS_. Indeed, controlling for the inbreeding effect of selfing did not fully account for the actual reduction of *N*_e _caused by selfing. Munoz *et al*. (Munoz F, Violle C, Cheptou P-O: Plant mating system is related to CSR strategy: from selfing ruderals to outcrossing competitors. Unpublished) demonstrated that selfing species are mainly early-successional species, suggesting that metapopulation dynamics could differ depending on the mating system.

Despite the strong impact of the mating system on *F*_ST_, *F*_IS _rather than *t*_m _is the main explanatory variable of *F*_ST_, as already apparent in our previous study [6]. However, this difference is not significant, since identical results were obtained in partial regression analyses regardless of which factor, *t*_m _or *F*_IS_, is first included in the model (data not shown). The important point here is that both *t*_m _and *F*_IS _explain the *F*_ST_, showing that they contain complementary information. The outcrossing rate *t*_m _is expected to be an accurate predictor of species inbreeding history only if outcrossing rate is stable over time, which is generally not the case. By contrast *F*_IS _reflects inbreeding not only in the current generation but also in previous generations [58]. Furthermore, *F*_IS _integrates other sources of inbreeding besides that caused by selfing, such as intra-population genetic structuring (Wahlund effect, [59]) resulting in biparental inbreeding and/or selection against inbreds (inbreeding depression).

Although plant traits are not directly associated with *F*_ST_, they could still control it indirectly, via their effect on mating system and on inbreeding. This had not been tested in previous studies because accurate quantifications of mating system were missing. Here we show that there is a strong association between mating system and perenniality: in contrast to short-lived plants, most long-lived plants are outcrossed. Growth form also appears to play a role on mating system, because woody plants (which are all long-lived) are more outcrossed than perennial herbaceous plants. Interestingly, plant stature does not influence outcrossing rate once perenniality and growth form are controlled for. Finally, we did not find any association between mating system and mode of pollination, in contrast to previous studies that did not use phylogenetic corrections [20,22,60]. Overall, the main predictor of outcrossing rate is perenniality, suggesting that there is a repeated pattern in unrelated lineages of evolutionary change towards selfing driven by short life cycles (or reciprocally an evolutionary transition towards outcrossing driven by longer life cycles). This result points to selective forces driving the evolution of plant mating systems [61].

Perenniality is related not only with mating system but also with inbreeding. Long-lived species, which are more outcrossed than short-lived species, have lower inbreeding coefficients. Interestingly, perenniality still influences the level of inbreeding *F*_IS _once mating system has been controlled for (i.e. a perennial species having the same outcrossing rate than an annual species tends to have less inbreeding). This might be explained by a difference between short-lived and long-lived species in their level of biparental inbreeding and/or of inbreeding depression. The first possibility implies that short-lived species are more subject to biparental inbreeding than long-lived ones, for instance due to reduced gene flow. Our data do not provide support for this hypothesis, as biparental inbreeding appears to be a general phenomenon in plant species. The second possibility implies that selection against inbreds is stronger in perennials than in non-perennials [17-19]. Our data confirm the trend of increased inbreeding depression from short-lived herbaceous to long-lived herbaceous and to woody species. Hence, *F*_IS _integrates information not only on selfing but also on selection against inbreds, which is associated with perenniality. This could explain why perenniality remains a significant explanatory variable of *F*_IS _once the outcrossing rate is accounted for (table 1). Similarly, inbreeding depression might explain why *F*_IS _remains a significant explanatory variable of *F*_ST _once the outcrossing rate is accounted for (table 1).

Two opposite scenarios can be proposed to explain the negative relationship between inbreeding depression and *F*_ST_. Selection against inbreds could lower *F*_ST _by reducing the effective selfing rate and hence intra-population drift. Alternatively, genetic structure could affect inbreeding depression. A plant in a given population will receive pollen either (i) from a pollen donor from another population (external-outbred pollen, P_eo_), (ii) from a pollen donor from the same population (local-outbred pollen, P_io_), or (iii) from its own pollen (self-pollen, P_s_). Assuming that there is no outbreeding depression, the offspring fitness should vary according to the pollen source, as follows: W_eo _> W_io _> W_s_. When *F*_ST _is low, one can expect W_io _to be close to W_eo _because external and local pollen will be genetically similar. When *F*_ST _is large, one can expect W_io _to be close to W_s _because individuals are strongly related and the population will have already been purged from partially recessive deleterious alleles. This would result in lower inbreeding depression δ in short-lived, high-*F*_ST _species.

The high outcrossing rate found in woody perennial is likely a strategy to avoid the deleterious effects of inbreeding. The reason why woody perennials are particularly sensitive to inbreeding remains elusive. There are two main hypotheses, one stating that deleterious effects become magnified through time, due to their multiplicative effects on fitness [18], and the other that deleterious effects depend on the number of cell divisions per cycle [17]. As cell divisions accumulate through time, the two hypotheses are difficult to distinguish. Moreover, stature is certainly a poor surrogate for the number of cell divisions [17], whereas perenniality is a poor surrogate for generation time. Nevertheless, our results do suggest that the association between stature and mating system could be a by-product of the correlation of stature with perenniality and growth form. This is illustrated by the absence of association between stature and *t*_m _among herbaceous species and among woody species.

## Conclusion

Despite imprecision associated with the measures of *F*_ST_, *F*_IS _and *t*_m_, our study has revealed general macroevolutionary patterns emerging from the phylogenetically-controlled correlation structure of these measures with species traits. We confirm that mating system is the main determinant of *F*_ST_, whereas its impact on *F*_ST_' suggests that some degree of selfing eventually reduces pollen-mediated gene flow. The effect of selective processes associated with selfing can not be fully disentangled but could also play a role. Selfing and mating between relatives also affect *F*_ST _by increasing inbreeding, which enhances genetic drift. However, other processes can affect inbreeding, in particular inbreeding depression: long-lived species present higher inbreeding depression than short-lived species. Perenniality, and, to a lesser extent, woodiness (which are both surrogates of generation time) appear to have a major influence on plant mating system and inbreeding and hence indirectly on plant genetic structure. These results, together with previous ones showing that generation time affects the rate of molecular evolution [62,63], point to the complex inter-relation between life history traits and plant evolution.

## Authors' contributions

Study design and collection of data were done by RJP and JD. Statistical analyses were performed by JD. Decomposition of population genetic formula (*F*_ST(δ)_) was done by OJH. All three authors have been involved in drafting and revising the manuscript.

## Supplementary Material

Additional file 1 **Plant traits and genetic characteristics of the species**. Description of the set of variables studied for the set of species. Click here for file

Additional file 2 **Phylogenetic supertree of the 263 species**. This figure describes the topology of the phylogenetic tree used for PICs analyses. Click here for file

Additional file 3 **Mean and standard deviation of *t*_m_, *F*_IS _and *F*_ST _as a function of the various plant traits**. The data provided represent the mean and standard deviation of genetic characteristics of the studied species by plant traits categories (growth form, perenniality, mode of pollen dispersal, mating system). Click here for file

Additional file 4 **Phylogenetically-controlled regression analyses between *F*_ST_, *F*_ST_' and other variables**. The data provided represent the results of the PICs analyses among *F*_ST _and *F*_ST_' and other variables for short-lived, long-lived and woody species separately. Click here for file

Additional file 5 **Mating system distribution (percentage of species) in function of species perenniality, growth form and mode of pollen dispersal**. The data provided represent the distribution of the outcrossing rates among all species by perenniality, growth form and mode of pollen dispersal categories. Click here for file
