# Supplementary material for: Plant traits correlated with generation time directly affect inbreeding depression and mating system and indirectly genetic structure
Source: BMC Evol Biol. 2009 Jul 27;9:177. doi: 10.1186/1471-2148-9-177 (PMC2728730; doi:10.1186/1471-2148-9-177)
Supplement: Additional file 1 — Plant traits and genetic characteristics of the species. Description of the set of variables studied for the set of species. [file 1471-2148-9-177-S1.doc]

**Additional file 1** : Plant traits and genetic characteristics of the species.
