# Supplementary figures and images for: Plant traits correlated with generation time directly affect inbreeding depression and mating system and indirectly genetic structure
Source: BMC Evol Biol. 2009 Jul 27;9:177. doi: 10.1186/1471-2148-9-177 (PMC2728730; doi:10.1186/1471-2148-9-177)

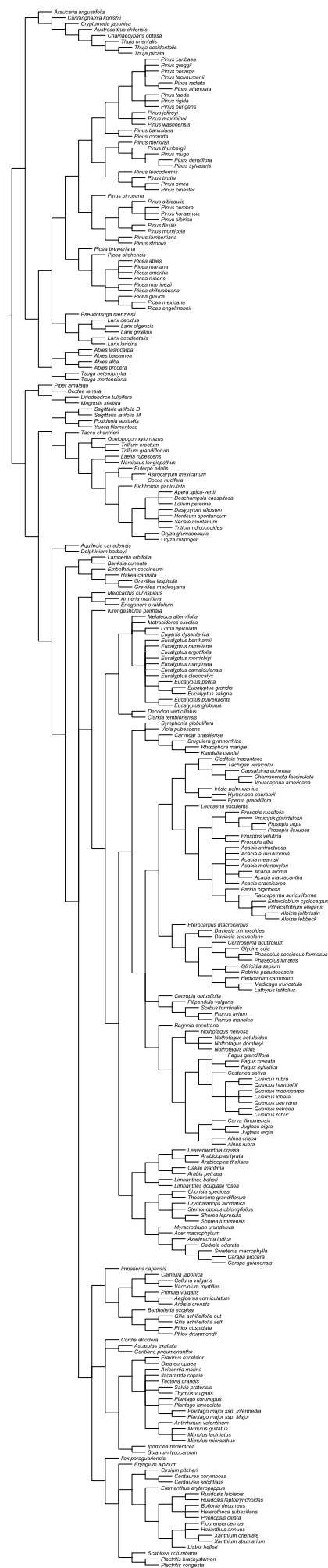

Supplement: Additional file 2 — Phylogenetic supertree of the 263 species. This figure describes the topology of the phylogenetic tree used for PICs analyses. [file 1471-2148-9-177-S2.pdf]
