# Supplementary material for: Plant traits correlated with generation time directly affect inbreeding depression and mating system and indirectly genetic structure
Source: BMC Evol Biol. 2009 Jul 27;9:177. doi: 10.1186/1471-2148-9-177 (PMC2728730; doi:10.1186/1471-2148-9-177)
Supplement: Additional file 3 — Mean and standard deviation of tm, FIS and FST as a function of the various plant traits. The data provided represent the mean and standard deviation of genetic characteristics of the studied species by plant traits categories (growth form, perenniality, mode of pollen dispersal, mating system). [file 1471-2148-9-177-S3.doc]

**Additional file 3:** Mean and standard deviation of *t*_m_, *F*_IS_ and *F*_ST_ as a function of the various plant traits

|  |  | *t*_m_ | *F*_IS_ | *F*_ST_ | *N* |
| --- | --- | --- | --- | --- | --- |
| **Growth form** | H | 0.585 ± 0.341 | 0.271 ± 0.348 | 0.284 ± 0.202^A^ | 77 |
|  | W | 0.860 ± 0.171 | 0.064 ± 0.156 | 0.127 ± 0.109 | 186 |
| **Perenniality** | SL | 0.472 ± 0.373 | 0.408 ± 0.402 | 0.347 ± 0.231 | 32 |
|  | LL | 0.822 ± 0.215 | 0.085 ± 0.191 | 0.148 ± 0.131 | 231 |
| **Growth form / Perenniality** | H/SL | 0.456 ± 0.365 | 0.415 ± 0.396 | 0.351 ± 0.229 | 31 |
|  | H/LL | 0.671 ± 0.296 | 0.174 ± 0.276 | 0.239 ± 0.169 | 46 |
|  | W/LL | 0.860 ± 0.171 | 0.064 ± 0.156 | 0.127 ± 0.109 | 186 |
| **Pollination mode** | A | 0.837 ± 0.244 | 0.083 ± 0.214 | 0.115 ± 0.131 | 102 |
|  | B | 0.743 ± 0.271 | 0.150 ± 0.264 | 0.209 ± 0.165 | 161 |
| **Mating system** | MS | --- | 0.150 ± 0.227 | 0.189 ± 0.156 | 130 |
|  | AS | --- | 0.029 ± 0.126 | 0.126 ± 0.111 | 122 |

^A^ Mean ± standard error
