# Supplementary material for: Plant traits correlated with generation time directly affect inbreeding depression and mating system and indirectly genetic structure
Source: BMC Evol Biol. 2009 Jul 27;9:177. doi: 10.1186/1471-2148-9-177 (PMC2728730; doi:10.1186/1471-2148-9-177)
Supplement: Additional file 4 — Phylogenetically-controlled regression analyses between FST, FST' and other variables. The data provided represent the results of the PICs analyses among FST and FST' and other variables for short-lived, long-lived and woody species separately. [file 1471-2148-9-177-S4.doc]

**Additional file 4. Phylogenetically-controlled regression analyses between *F*_ST_,*F*_ST_’ and other variables**

|  | Long lived species^a^ | | | |  | Herbaceous^b^ | | | |  | Woody^c^ | | | |
| --- | --- | --- | --- | --- | --- | --- | --- | --- | --- | --- | --- | --- | --- | --- |
|  | ***F*_ST_** | | ***F*_ST_’** | |  | ***F*_ST_** | | ***F*_ST_’** | |  | ***F*_ST_** | | ***F*_ST_’** | |
|  | R ^‡^ | ΔR² ^†^ | R ^‡^ | ΔR² ^†^ |  | R ^‡^ | ΔR² ^†^ | R ^‡^ | ΔR² ^†^ |  | R ^‡^ | ΔR² ^†^ | R ^‡^ | ΔR² ^†^ |
| *t*_m_ | -0.262*** | 0.069 | -0.209** | 0.044 |  | -0.511*** | -- | -0.398*** | 0.158 |  | -0.181* | -- | -0.136 ^NS^ |  |
| *F*_IS_ | 0.249*** | 0.028 | 0.100 ^NS^ |  |  | 0.528*** | 0.279 | 0.381*** | -- |  | 0.235*** | 0.055 | 0.083 ^NS^ |  |
| Stature | -0.143* | -- | -0.149* | -- |  | -0.241* | -- | -0.225 ^NS^ |  |  | -0.05 ^NS^ |  | -0.055 ^NS^ |  |
| Growth form^d^ | -0.158* | -- | 0.161* | -- |  | **NC** |  |  |  |  | **NC** |  |  |  |
| Perenniality^e^ | **NC** |  |  |  |  | -0.148 ^NS^ |  | -0.098 ^NS^ |  |  | **NC** |  |  |  |
| Pollination mode^f^ | 0.007^NS^ |  | 0.013 ^NS^ |  |  | 0.025 ^NS^ |  | 0.039 ^NS^ |  |  | 0.051 ^NS^ |  | 0.055 ^NS^ |  |

Same legend as table 1.
