# Supplementary material for: Plant traits correlated with generation time directly affect inbreeding depression and mating system and indirectly genetic structure
Source: BMC Evol Biol. 2009 Jul 27;9:177. doi: 10.1186/1471-2148-9-177 (PMC2728730; doi:10.1186/1471-2148-9-177)
Supplement: Additional file 5 — Mating system distribution (percentage of species) in function of species perenniality, growth form and mode of pollen dispersal. The data provided represent the distribution of the outcrossing rates among all species by perenniality, growth form and mode of pollen dispersal categories. [file 1471-2148-9-177-S5.doc]

**Additional file 4:** Mating system distribution (percentage of species) in function of species perenniality, growth form and mode of pollen dispersal. Each category of the three traits considered is represented by a half pie chart (180° equals 100%). Mixed mating species: 0.1<*t*_m_<0.9 (N=129); outcrossed species *t*_m_>0.9 (N=117); selfed species *t*_m_<0.1 (N=5).
